# Evaluating Heterogeneous Conservation Effects of Forest Protection in Indonesia

**DOI:** 10.1371/journal.pone.0124872

**Published:** 2015-06-03

**Authors:** Payal Shah, Kathy Baylis

**Affiliations:** 1 Okinawa Institute of Science and Technology Graduate University, Onna-son, Okinawa, Japan; 2 Department of Agriculture and Consumer Economics, University of Illinois at Urbana-Champaign, Champaign, Illinois, United States of America; Tennessee State University, UNITED STATES

## Abstract

Establishing legal protection for forest areas is the most common policy used to limit forest loss. This article evaluates the effectiveness of seven Indonesian forest protected areas introduced between 1999 and 2012. Specifically, we explore how the effectiveness of these parks varies over space. Protected areas have mixed success in preserving forest, and it is important for conservationists to understand where they work and where they do not. Observed differences in the estimated treatment effect of protection may be driven by several factors. Indonesia is particularly diverse, with the landscape, forest and forest threats varying greatly from region to region, and this diversity may drive differences in the effectiveness of protected areas in conserving forest. However, the observed variation may also be spurious and arise from differing degrees of bias in the estimated treatment effect over space. In this paper, we use a difference-in-differences approach comparing treated observations and matched controls to estimate the effect of each protected area. We then distinguish the true variation in protected area effectiveness from spurious variation driven by several sources of estimation bias. Based on our most flexible method that allows the data generating process to vary across space, we find that the national average effect of protection preserves an additional 1.1% of forest cover; however the effect of individual parks range from a decrease of 3.4% to an increase of 5.3% and the effect of most parks differ from the national average. Potential biases may affect estimates in two parks, but results consistently show Sebangau National Park is more effective while two parks are substantially less able to protect forest cover than the national average.

## Introduction

Approximately 16.3 million square kilometers of forests worldwide are protected to limit the conversion of forests to commercial activities [1]. The estimated effect of such protection is mixed, and varies greatly from one protected area to another [2–8]. One country where forest protection has been widely implemented is Indonesia. Indonesia is home to some of the most biologically diverse forests in the world, providing essential habitat for endangered species such as tigers, elephants and orangutans. Agricultural expansion and illegal logging has led to rampant deforestation, degraded ecosystems, habitat loss and increased carbon emissions. In response, between 1990 and 2010, Indonesia increased the total land area under protection from 10% to 14% [1]. However, the effect of this protection is likely to vary across Indonesia. In this article, we ask whether and where forest protection has been successful in preserving forest cover. We attempt to disentangle two possible sources of this variation or ‘treatment heterogeneity’ in the estimated effects of protection: true underlying differences in the effect of protection and spurious variation in the estimates driven by differing degrees of bias. Understanding where protection has succeeded and where it has failed to preserve forest can help policy makers more effectively design future conservation efforts across the country.

Placing land under protection does not guarantee a decrease in deforestation [9]. First, protected land may not be under threat of conversion (i.e. the forest under protection is not ‘additional’). Second, government protection may remove the local forest user’s incentive to regulate resource consumption, resulting in decreased monitoring and increased illegal logging or land conversion [10, 11]. Thus, particularly in a setting of uncertain land tenure and incomplete monitoring and enforcement, the effect of establishing forest protection is unclear.

One challenge when evaluating conservation policy is to derive an appropriate counterfactual: what would have happened in the absence of the policy [9]? Protected areas are seldom randomly distributed across the landscape [9]. To overcome the potential selection bias that arises from non-random location of protected areas, several empirical studies on forest conservation use matching methods that formally develop a counterfactual control group [2–8, 12–18]. Depending on what would have happened to that land in absence of the park, for example, would land clearing for agriculture have been permitted, one may need to control for the type of land use activity allowed in the set of controls [19]. Studies that use matching methods compare deforestation rates inside protected areas with relevant counterfactuals to evaluate the success of the protected areas in limiting deforestation while controlling for observable parcel level characteristics. Their results indicate that on average, protected areas have reduced deforestation, although often not as much as a naïve comparison would imply. Further, these studies do not consider the possible variation of effectiveness across space.

Several studies estimate the quantity and causes of deforestation in Indonesia [20–31]. These studies find a wide variation in the rates and trends of deforestation across regions in Indonesia owing to the differences in regional characteristics. This variation in levels of deforestation and effect of protection was likely exacerbated by the decentralization of much of the control over economic development and land use from federal to provincial and district governments during the *Reformasi* period of 1999–2002. The local demand for revenue raises concerns about the conversion of forests into large-scale industrial plantations, small-scale commodity-based agriculture [19], and mining [32–34]. In a recent study, [35] find that the increase in the number of districts in the post-Suharto regime is associated with an increase in deforestation activity between 2000 and 2008, pointing to forest losses from decentralization.

Similarly, studies find different effects of forest protection across Indonesia. In Sumatra, protected areas were found not to reduce deforestation any more than unprotected areas where logging is allowed but land conversion is prohibited [20]. In Kalimantan, protected lowland forests declined by 56% between 1985 and 2001 [24]. Between 1996 and 2002, over 2 million hectares of forest were lost in proposed and existing protected areas in Kalimantan [22]. In Sulawesi, Lore-Lindu National park is estimated to have reduced deforestation by more than 9% between 1983 and 2001 [36]. Each of these studies estimates the effects of protection in only some parks, making it difficult to compare the results across regions.

In recent work, some authors move beyond estimating a single treatment effect and explore how that treatment varies over space [3–5, 13–17, 37]. It is important to consider the spatial variation in policy impacts to guide effective conservation planning that balances the local cost of protection with local and global benefits [13, 14, 16]. The study by [5] uses a locally weighted scatter plot smoothing and a semi-parametric partial linear differencing model to explore the variation in the effect of protection on reduction in deforestation and poverty levels. They find the effect of protection on forest cover and poverty levels varies with baseline characteristics such as distance to major city and slope. In recent work, [38] use a general equilibrium framework to explain the differences in forest leakage in the vicinity of protected areas using observed variation in local economic conditions. They find substantial variation in the degree to which Indonesian protected areas increase or reduce nearby forest cover driven in large part by the demand elasticities of different forest and agricultural products competing for land in these regions.

Differences or similarities in treatment estimates across the various protected areas in Indonesia might be driven by different data-generating processes over space, which we would like to observe, and ideally to explain. However, the distribution of treatment estimates may equally be affected by differential levels of estimation bias over space. Imagine for example that establishing a protected area in two provinces has exactly the same true effect on forest cover change, but that in one province, many similar parcels exist outside the park that act as appropriate controls, whereas in the other province, all of the controls face high pressure for forest loss, biasing the estimated effect of protection upward. This difference in deforestation pressure in the second province’s control parcels might arise from the location of the protected area being associated with unobservables that are also associated with lower rates of deforestation. For example, the protected area may be placed in a particularly inaccessible location. Alternatively, the difference in deforestation pressure could be driven by ‘bad’ matches, where the available control parcels in the region exhibit much worse covariate balance than in the first province. At first glance, it may appear as if the second province has a much more successful protected area strategy, but all that occurred is that the treatment estimate is more accurate in the first province than the second. Along with creating the false appearance of variation in protection, differential bias might instead mask true variation in the regional effects of parks.

In this study, we first estimate the effect of the different parks on forest conservation and then estimate the same effects controlling for different sources of potential bias to observe which regional differences persist across the alternative estimation methods. We begin by estimating the effects of the protected areas using matching methods combined with a difference-in-differences approach, also known as Before-After-Treatment-Intervention (BACI), where we match parcels over their own characteristics as well as the characteristics of their contiguous neighbors to generate our control group [6, 8]. We show that a single average treatment effect estimate masks a great deal of variation in the effectiveness of protection. Next, we explore whether and where the differences in estimated effect of protection persist after controlling for potentially bad matches and testing for sensitivity to bias driven by unobservables. Last, we use a conditionally parametric locally weighted regression (CPARLWR) to estimate how parcel characteristics jointly vary in their impact on forest cover change across space. This approach allows the estimated data generating process to vary across space, thus highlighting how observable characteristics differ in their effect on deforestation and limiting potential bias generated by spatially-correlated unobservables. We compare the results across these various approaches to identify those protected areas that truly exhibit different effectiveness from the national average.

This article makes several contributions to the existing literature on the impact evaluation of protected areas. Only a few papers explicitly estimate how the effect of protection varies, and those do not consider differences in potential bias that might drive the differences in estimates. Second, we propose a process that allows researchers to explore the source of estimated variation in treatment. Last, we use a novel CPARLWR method to estimate differential deforestation pressure across space and thus different effectiveness of protected areas. We find substantial variation in effect across the seven studied protected areas. Even after controlling for potential bias, several parks appear to be less effective than average while two others are significantly more effective than average. We find the effects of protection vary not only across the different parks of Indonesia but even within some of the larger national parks such as Kerinci Seblat. These different outcomes may suggest the need for targeted intervention on the part of the government to improve overall protection outcomes.

## Background and Setting

### Study Area

Indonesia covers a total area of 1,904,569 square kilometers and is broadly divided into five island-regions, Java, Sumatra, Kalimantan, Sulawesi and Papua, 33 provinces and approximately 500 districts. Indonesia has the third highest rate of annual forest loss in the world. The total primary forest cover loss between 2000 and 2012 is estimated at 6.02 million hectares [39]. In response, the Indonesian government increased the terrestrial area under protection to 14% by 2010 from 10% in 1990, comprising a total area of 2.7 million hectares. We use data for seven new national parks that were established in Indonesia after 1999 comprising an area of 23,275 square kilometers (1.2% of the total land area). Previous studies have used a wide variety of spatial resolution including uniform and non-uniform parcel size for estimating the effect of protection [8;18;20;40–43]. We follow [42] and use a uniform 3 km by 3 km grid with measures of percent change in forest cover, protection status and biophysical characteristics of land for a total of 195,466 parcels. Our measure of interest is the percent of these parcels covered by primary forest.

These protected areas were primarily established to protect habitat for endemic flora and fauna. Table 1 provides details about the year of establishment, size and number of 3 km by 3 km parcels for each of the seven national parks we study. Three national parks were established in Sumatra to conserve the quickly deteriorating lowland forests in the region. Kerinci Seblat National Park, the largest national park in Sumatra, was officially gazetted in 1999, though its park boundary was delineated in the 1980s [44]. Studies find the presence of logging concessions in neighboring areas and forest conversion to farmland both inside and outside the national park boundary [45]. Batang Gadis and Tesso Nilo national parks were established in Sumatra in 2004. Illegal logging, expanding agriculture, and encroachment continue to cause deforestation in the lowland forests of Batang Gadis and Tesso Nilo National Park [46, 47].

**Table 1:** National Parks in Indonesia established after 1999.

National Park	Year of Establishment	Area (sq. km.)	No. of Observations
**Kerinci Seblat**	1999	13,750	1801
**Batang Gadis**	2004	1,080	148
**Tesso Nilo**	2004	386	56
**Gunung Ciremai**	2004	155	33
**Sebangau**	2004	5,687	711
**Bantimurung Bulusaraung**	2004	480	87
**Aketajawe Lolobata**	2004	1,673	189

Of the three national parks established in Java in 2004, we only study Gunung Ciremai National Park, a relatively small volcanic area designed to protect the biodiversity in the region. The area is under threat from many legal and illegal volcanic sand mining companies [48]. Sebangau National Park was established in Kalimantan in 2004 in an area that is predominantly peat forest and was previously the site of substantial illegal logging and a failed mega rice project Sebangau National Park is home to Bornean orangutans, proboscis monkeys and Bornean gibbons [49]. Bantimurung Bulusaraung National Park was established in 2004 in Sulawesi and includes the second largest karst area in the world. Aketajawe Lolobata National Park was established in 2004 in the Papua region of Indonesia with a primary objective of protecting endemic bird species [50]. According to [50], this particular national park and the surrounding area are under increasing pressure from the opening of new mines such as the Weda Bay nickel mine.

### Forest Cover Data and Identification Strategy

Our response variable is continuous and represents the change in primary forest cover within 3 km by 3 km parcels between 2000 and 2012. The forest cover data are based on a study by [39] that provides estimates of primary forest cover for Indonesia between 2000 and 2012. The data illustrate clear spatial variation in loss of forest cover across the different regions of Indonesia. Java accounted for 0.2% of the decrease in forest cover, Sumatra accounted for 47.9% of the decrease, Kalimantan accounted for 40% of the decrease, Sulawesi accounted for 6.3% of the decrease and Papua accounted for 5.6% of the decrease. We use data available from the World Database on Protected Areas to identify 3,057 3 km by 3 km parcels that were established as national parks between 1999 and 2012 and consider these parcels as “treated”. The protected areas are managed by the directorate general of conservation (PHKA) under the Ministry of Forestry (now the Ministry of Environment and Forestry). We exclude 21,510 3 km by 3 km parcels that were designated as protected prior to 1999 from our analysis. While our forest cover data only begin in 2000, we include parcels associated with Kerinci Seblat National Park that was formally established in 1999 because it is likely that the impact of establishing a protected area on changes in forest cover takes more than a few months to take effect. The final dataset consists of 3,057 treatment parcels and 170,899 eligible control parcels across the whole of Indonesia. Table 1 provides additional details about the size and number of treatment observations for each of the seven national parks.

We obtain data on covariates such as slope, elevation, distance to roads, distance to rivers, distance to cities, peat depth, and administrative boundaries for all treated and eligible control parcels [51]. The summary statistics for the change in parcel-level primary forest cover between 2000 and 2012 and these covariates for treated and eligible control parcels are provided in S1 Table for each park.

## Methods

We estimate the average treatment effect on the treated (ATT), which is a measure of the impact that the establishment of a protected area has on the change in forest cover within the park. Our measure of ATT is reported in percentage change in forest cover within each 3 km by 3 km (i.e. 900 hectares) parcel from 2000 to 2012.

If protection was randomly allocated across land parcels, one could estimate the ATT by comparing forest cover change inside and outside protected areas before and after the establishment of the protected area. This simple difference-in-differences approach controls for time-invariant unobservable characteristics. However, the location of protection is not random, as is evident from the difference in covariates in treatment versus control parcels (S1 Table). These covariates may affect the pressure for deforestation, thus, we would expect the change in forest cover to differ inside and outside protected areas even if protection had no effect. To address this concern, we generate a comparable set of control observations using matching methods to evaluate the impact of protection [52, 53]. Matching is an *ex post* identification technique that uses observable characteristics to identify a counterfactual group from land parcels that are not protected and that are similar to the treatment group [54, 55]. If one can identify observable characteristics such that any two parcels of land with the same characteristics portray identical responses to protection, then the estimated treatment effect is said to be unbiased. An unbiased measure of ATT thus requires that the observable variables used to identify the counterfactual capture all characteristics that jointly affect selection into protection and forest cover change.

In the matching process, we include all covariates that are likely to affect the selection of a parcel into protection and the pressure for deforestation. Indonesian protection policy recognizes land parcels located at certain slopes and elevation that have sensitive soil types and greater peat land depth as areas that qualify for protection [56]. Previous literature that models land-use decisions identify plot-level accessibility characteristics such as elevation, slope, distance to roads, distance to rivers, distance to nearest city and land use opportunities as important determinants of forest clearing [57–59]. We use all covariates identified in S1 Table to select appropriate counterfactuals for the treated parcels. To control for differences in local policy, we ensure that matched parcels are selected from the same province and eco-region as treatment parcels. We use OLS regressions to check for the relationship between the covariates and the change in forest cover on all non-protected parcels. We find that the average distance from city, roads, elevation and slope have a significant positive effect while the extent of forest cover in 2000 and peat depth has a significant negative effect on forest cover change. We also find significant fixed effects for many provinces and ecoregions.

Previous studies find evidence of spatial dependence in forest cover change and land use models [59, 60–63]. We test for spatial autocorrelation in the change in forest cover using the Moran’s I test based on a first order queen’s contiguity weights matrix. The first order contiguity matrix captures the intuition of how changes in forest cover vary over the landscape, with logging equipment being easier to move across a continuous space, and logging roads making contiguous parcels more accessible. We find evidence of significant positive correlation of 0.67 (with a p-statistic of <0.0001) between forest cover change on a given parcel of land and that on its immediately contiguous neighboring parcels and thus we find counterfactual parcels that are similar to treatment parcels based on their own characteristics as well as the characteristics of neighboring parcels [8]. We select a one-to-five nearest neighbor covariate matching with replacement using a generalized version of the Mahalanobis distance metric implemented in R [53]. We use the bias adjustment to correct for the differences in the covariates for each matched pair using the estimated coefficients from a linear regression of the covariates on the expected outcome. We also estimate the variance at all observations to address the problem of potentially heteroskedastic error terms [64].

We provide ATT estimates at two levels: for Indonesia as a whole and for each of the seven new national parks. We then test whether the ATT estimates for the individual parks differ from the national ATT using a two-sample t-test for difference in population means with unequal variances.

Next, we want to explore whether these differences in estimated effect of protection come from true underlying variation in protection or different precision in the estimates. We first explore the potential bias coming from differences in the precision of each park’s counterfactual. To assess the quality of the matches, we check the covariate balance for each park, testing the normalized differences in covariate means, and their distribution. The normalized difference in mean is the difference in the average covariate value divided by its standard deviation [65]. We test for differences in the distribution using eQQ plots that graph the covariate values in the same quantile of the treated against those in the control, allowing us to observe if characteristics are distributed similarly across both treatment and control groups [66].

Second, we attempt to remove possible bias driven by differences in the quality of matches by conducting caliper matching that sets the same tolerance level for matches across the different parks. Caliper matching drops all treated parcels for which the matching routine cannot find good matches (i.e. matches whose covariate values are not within a predefined standard deviation of the covariates of treatment observations). We compare the results with and without caliper matching to explore to what degree ‘bad matches’ in one region are driving the variation in our results across parks.

As noted above, matching methods are not robust against “hidden bias” arising from the existence of unobserved variables that simultaneously affect assignment to treatment and outcome. To explore whether differences in treatment estimates may be driven by this hidden bias, we estimate Rosenbaum bounds [67] by park. Rosenbaum bounds provide a measure of how strongly an unobservable covariate could affect the estimated treatment effect by estimating test statistics for different levels of the odds ratio, Γ, where a higher odds ratio is associated with unobservables playing a larger role in the selection of treatment parcels. The bounds identify the odds ratio at which the estimated treatment effect is no longer significantly different from the ATT for the whole of Indonesia (Γ_1_) or zero (Γ_2_). Thus, a higher Γ implies that the estimated results are robust against a greater potential selection bias, while a low Γ implies that even a mild selection bias could make the estimate insignificant (where Γ = 1 indicates that no hidden bias exists).

We use the *rbounds* package in Stata to estimate two separate odds ratios: 1) Γ_1_ measures the degree to which unobservables may affect whether the park ATT estimate is significantly different than the national ATT estimate, and 2) Γ_2_ provides a measure of how robust the park-level ATT estimate is to the effect of unobservable variable(s) on the selection into treatment. We identify Γ_1_ as the lowest odds ratio that contains the national ATT estimate between the upper and lower bounds of the Hodges-Lehman point estimate for each park. We estimate Γ_2_ based on the Wilcoxon’s sign rank test for which the ATT estimates are robust against “hidden bias” at the 10% significance level. We calculate these odds ratios, Γ_1_ and Γ_2_, for each national park.

Once we have identified the source of variation in our treatment effects that may result from different degrees of bias, we turn to exploring the variation in estimated park effectiveness that may result from differences in economic pressure on forest use and other factors affecting the regional data generating process. We first control for potential differences in effectiveness that result from the type of economic activity in protected and unprotected parcels. Differences in the estimated effectiveness of parks may result from higher demand for certain land conversion activity in some regions rather than others. A previous study finds differences in the estimated effectiveness of protected areas when those protected areas are compared against counterfactuals where land use regulations prohibit conversion activity but allow for production activities such as commercial logging versus where those counterfactuals whose regulation includes all deforestation activity [19]. We estimate an additional measure of ATT that restricts the selection of counterfactual parcels to come from only those unprotected parcels that are sanctioned by the Indonesian government as suitable for limited production activities. For this estimation, we eliminate those parcels where land conversion activities are allowed. Effectively, this estimate captures how much forest cover parks protect compared to “production zone” areas, where sustainable commercial forest activities are allowed. Thus, we should be able to determine if some parks are particularly effective in protecting against local demands for land conversion but not against deforestation resulting from commercial forest activity. Under Indonesia's 1990 National Spatial Plan, the “production zone” includes areas allocated for commercial logging where deforestation is prohibited but selective logging that leads to sustainable forest use is allowed. The “conversion zone” includes regions allocated to industrial plantations, smallholder agriculture, mining, urban areas, and government-sponsored transmigration settlements.

True differences in treatment effects are likely driven by differences in data generating processes among parks. To estimate how the true data-generating process varies across space, one can use a multivariate locally-weighted regression (LWR). A multivariate LWR estimates a different coefficient for each variable and each observation by weighting each observation in a regression based on spatial proximity. One challenge with this approach is that one needs to impose some structure on the coefficients to be able to estimate the full set of coefficients [68]. We use the CPARLWR that imposes a parametric structure on the independent variables but allows this parametric regression to vary based on geographic coordinates. CPARLWR models local geographic variation in the data generating process using a flexible trend that varies across space based on geographic distances. As shown in [69], the model specification for the CPARLWR is,

$$y_{i}\;=\;\beta {\left({lo}_{i},{la}_{i}\right)}^{'}x_{i}+u_{i}$$ (1)

Here, *y* represents the change in forest cover, *x* represents the vector of independent variables including a treatment dummy and the geographic covariates that affect forest cover change decisions and *u* is the error term. The terms *lo* and *la* represent the geographic coordinates for each observation *i*. The coefficients, *β*, are assumed to vary smoothly over space. The coefficients at a target location are estimated as the weighted least squares,

B^lo,la = ∑j = inwjxjxj'-1∑j = inwjxjyj (2)

where *w*_*j*_ represents a weight function.

We use the CPARLWR approach to calculate coefficients for the treatment dummy and the covariates for each observation in the matched dataset. This method enables us to extract the heterogeneous effects of protection and other covariates on forest cover change across the different parks. This method may also reduce the potential bias found in the earlier ATT estimates to the extent that unobservables are spatially correlated. We calculate the coefficient values for each observation within the seven national parks and Indonesia as a whole and then report the median coefficient as an estimate of the marginal effect of protection and other covariates on forest cover change. We map the coefficient estimates for each of the different parks to identify the extent of variation in treatment effectiveness and impact of other covariates on forest cover change within a park. As a measure of goodness of fit, we estimate the pseudo R^2^ using the fitted values for each parcel based on the CPARLWR estimates.

## Results

Results from the spatial matching model indicate that, on average, protected areas across Indonesia lost 0.7% less forest cover compared to similar non-protected areas over the 12-year period. The national ATT estimates are relatively robust to the number of matches used and the exclusion of parcels adjacent to protected areas as controls. Post matching, the normalized difference for all covariates is less than 0.25 standard deviations and the average normalized difference across all covariates is 0.09, as shown in Table 2. This statistic suggests that we are able to find a sufficient number of unprotected parcels that are similar to protected parcels based on the covariates included in the matching process [65].

**Table 2:** Indonesia and Park Level Results.

National Park	ATT in % for change in primary forest cover (Std. Errors)^1^	Avg. Norm. Diff. between treated and matched covariate values^2^	t-Statistic for Difference in Means between individual parks ATT and ATT estimate for Indonesia (Std. Errors)^3^	Gamma 1^4^	ATT in % for change in primary forest cover with Calipers (Std. Errors)^5^	ATT in % for Restricted Matching without including land sanctioned for conversion (Std. Errors)^6^	Median CPARLWR Coefficient (Minimum and Maximum values)^7^
**Indonesia**	0.71%	0.09	-	-	0.50%	0.01%	1.12%
	(0.43%)		-		(0.29%)	(0.41%)	(-3.68%, 5.49%)
**Gunung Ciremai**	0.05%	0.21	71.70	6.0	0.01%	0.05%	-0.02%
	(0.12%)		(0.0823)		(0.10%)	(0.12%)	(-0.02%, 0%)
**Batang Gadis**	0.67%	0.18	4.00	1.6	0.44%	0.73%	-1.26%
	(0.30%)		(0.0950)		(0.28%)	(0.30%)	(-1.57%, -0.96%)
**Tesso Nilo**	-2.69%	0.23	13.41	1.3	-2.69%	-1.71%	-3.40%
	(4.65%)		(2.2811)		(4.65%)	(4.58%)	(-3.68%, -3.09%)
**Kerinci Seblat**	1.05%	0.17	75.07	2.0	0.96%	1.02%	0.73%
	(0.33%)		(0.0406)		(0.28%)	(0.33%)	(-0.11%, 3.15%)
**Sebangau**	4.18%	0.20	130	1.1	5.10%	2.12%	5.25%
	(1.76%)		(0.2436)		(0.72%)	(1.60%)	(5.16%, 5.49%)
**Bantimurung Bulusaraung**	0.43%	0.20	49.2	5.3	0.40%	0.39%	1.99%
	((0.11%)		(0.0508)		(0.10%)	(0.10%)	(1.58%, 2.48%)
**Aketajawe Lolobata**	0.78%	0.25	8.31	2.3	0.69%	0.63%	0.82%
	(0.26%)		(0.0746)		(0.23%)	(0.23%)	(0.81%, 0.83%)

^1^These ATT estimates are based on the nearest neighbor covariate matching that includes the average covariates of neighboring parcels.

^2^The average normalized differences between matched and treatment parcel covariates is the average value of the absolute difference in mean values of the covariates divided by their standard deviations.

^3^The t-statistic is based on the t-test for difference in means with unequal variance between the national ATT estimate and the individual park level ATT estimate. All t-statistics are significant at the 1% level.

^4^Gamma 1 represents the lowest odds ratio based on the Hodges-Lehmann point estimates that includes the national ATT estimate of 0.71% of change in forest cover within protected areas in the upper and lower bounds of the point estimates.

^5^These ATT estimates are based on the nearest neighbor covariate matching that includes the weighted covariates and restricts covariates of matched parcels to be within one standard deviation of the covariates for treated parcels.

^6^These ATT estimates are based on the nearest neighbor covariate matching that only selects counterfactuals from unprotected parcels that are allocated for both protection or production activities under Indonesia’s 1990 Spatial Plan and where conversion is prohibited.

^7^These estimates represent the median coefficient estimates for the marginal effect of treatment on change in forest cover for all parcels that are within the national park boundaries.

**Fig 1:**
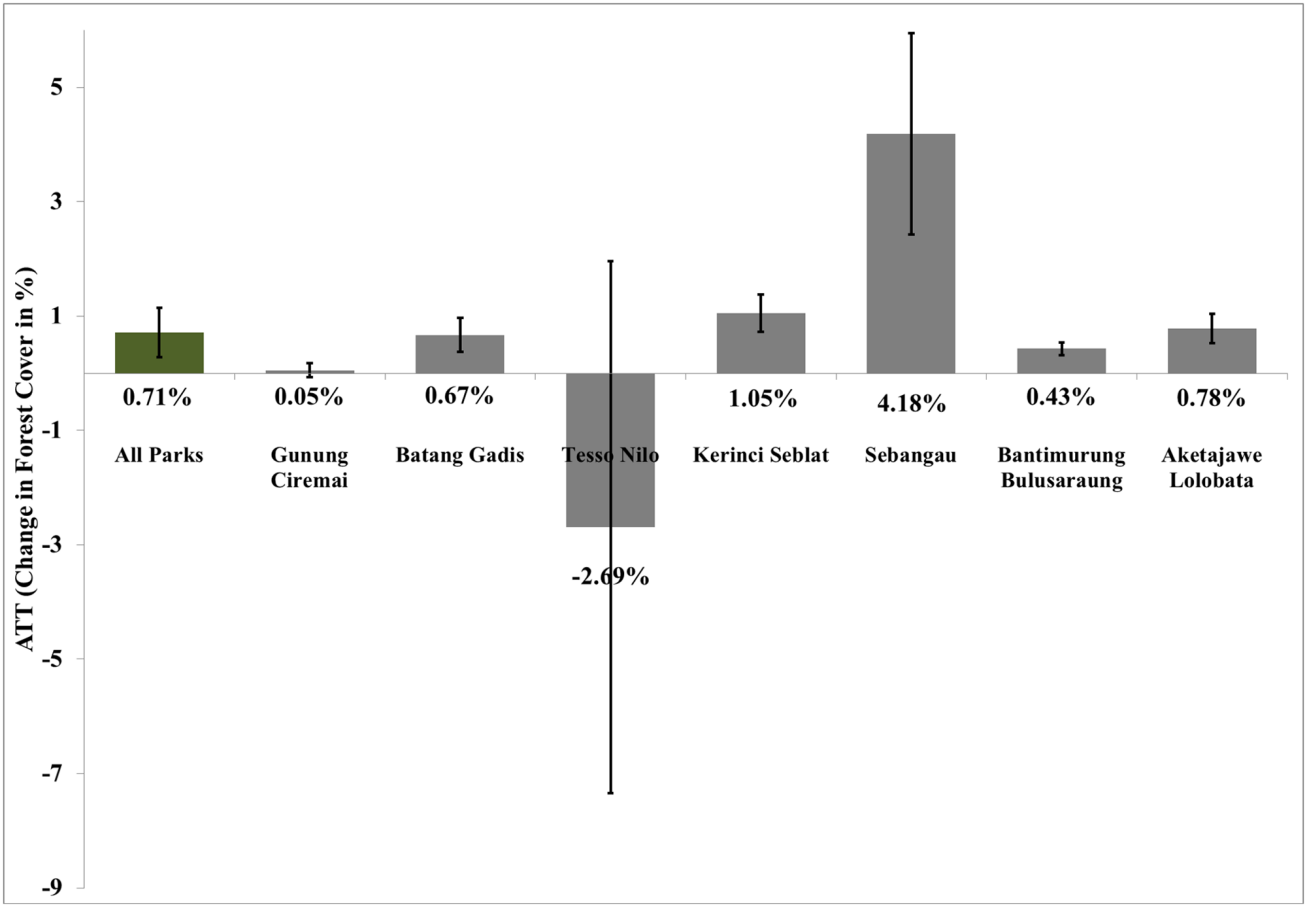
ATT for Indonesia and Seven National Parks. Note: This figure shows the ATT estimates based on the nearest neighbor covariate matching with replacement for all seven parks together as well as individually for each park. The percentage numbers indicate the ATT estimate in hectares divided by the total area of each parcel (i.e. 900 hectares). The error bars represent the standard errors for the ATT estimates.

However, these ATT estimates for Indonesia mask a great deal of variation in the effectiveness of individual protected areas in different parts of the country. Fig 1 illustrates the ATT estimates based on the spatial matching method for the seven new protected areas. Sebangau National Park has a high positive and significant ATT estimate indicating higher effectiveness at limiting forest cover loss within park boundary compared to the national estimate. Kerinci Seblat, Batang Gadis and Aketajawe Lolobata National Park’s ATT estimates are relatively similar to the national level estimates. ATT estimate for Gunung Ciremai National Park indicates very low and insignificant decrease in forest cover inside protected area boundaries. For one park, Tesso Nilo, we find an insignificant yet 2.7% more primary forest cover *loss* within the national park boundary than in the control observations. The ATT estimates for Gunung Ciremai, Kerinci Seblat, Batang Gadis, Aketajawe Lolobata and Bantimurung Bulusaraung National Park are robust to the variation in the number of matches used (i.e. anywhere from one to five matches) whereas results for Sebangau and Tesso Nilo National Parks are sensitive to the number of matches used in the matching routine. The estimates of Γ_2_ (shown in S4 Table through S10 Table) indicate that ATT estimates for Gunung Ciremai, Tesso Nilo, and Aketajawe Lolobata National Parks are less robust to possible hidden bias and the potential effect of unobservable(s) on selection into treatment. Covariate balance results (shown inS4 Table through S10 Table), also indicate higher normalized differences between matched and treated covariates for Aketajawe Lolobata National Park with an average normalized difference of 0.25. Thus, its treatment estimate should be taken with care. The average normalized differences for all other parks are less than 0.25 as shown in Table 2.

T-tests indicate that the park ATT estimates are significantly different from the national ATT estimate at a 1% significance level for all seven parks (column 3 of Table 2). ATT estimates for individual parks are not sensitive to caliper matching where we drop matches with covariate values that are more than one standard deviation away from covariate values for treated observations. However, as we increase the strictness level and use smaller calipers, ATT estimates for Sebangau National park shrink, indicating a smaller estimate of protected area effectiveness. Thus, less appropriate matches may exacerbate variation in the initial ATT estimates.

We use the Rosenbaum bounds to understand which parks have ATT estimates that are truly different from the national average, and which differences may be possibly driven by hidden bias in the ATT estimates. Some parks, such as Sebangau National Park, are more sensitive to this hidden bias with odds ratio, Γ_1_, of 1.1. The heterogeneity in the ATT estimates for Sebangau National Park may be largely driven by an increased probability that unobservables determine the choice of treatment. Alternately, we find that the extent to which the ATT estimates for Gunung Ciremai, Batang Gadis, Kerinci Seblat, Bantimurung Bulusaraung and Aketajawe Lolobata National Park are different from the national ATT estimate are relatively less sensitive to confounding variables with odds ratio of 6, 1.6, 2, 5.3 and 2.3 respectively. Table 2 summarizes these results. These results imply that ATT estimates for Gunung Ciremai, Batang Gadis, Kerinci Seblat, Bantimurung Bulusaraung and Aketajawe Lolobata National Parks are relatively less prone to hidden bias and the heterogeneity may instead be driven largely by true differences in effectiveness.

ATT estimates that restrict the matching to select control parcels only from the production zone find protected areas for the whole of Indonesia have less effect than a comparison to all allowed land uses, as shown in the second column of Table 2. Intuitively, we expect the ATT estimates that use counterfactuals only from the production zone to be lower than the ATT estimates that select counterfactual parcels from both production and conversion zones, since the first estimate captures the additional effect of protection over and above regulations that prohibit land conversion. We find this expected result for the whole of Indonesia, as well as for Sebangau, Bantimurung Bulusaraung and Aketajawe Lolobata National parks where the ATT estimates from the restricted matching are lower than the ATT estimates that include counterfactuals from both production and conversion zones (as shown in column 6 of Table 2). For other parks, estimates remain relatively unchanged.

**Fig 2:**
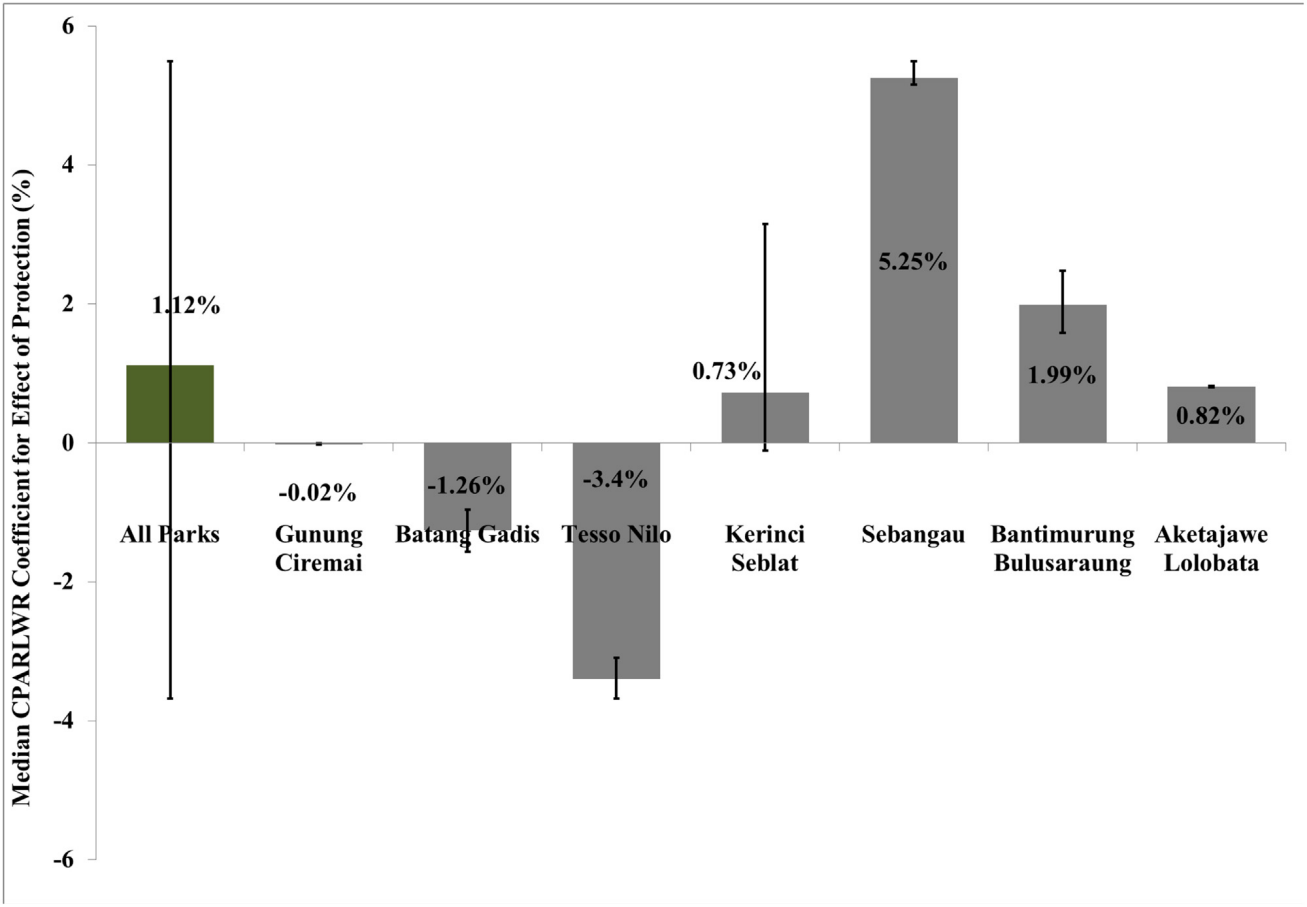
Average CPARLWR Treatment Effects for Indonesia and Seven National Parks. Note: This figure shows the average CPARLWR coefficient estimates for the treatment dummy for all seven parks together as well as individually for each park. The percentage numbers indicate the overall percentage gain or loss in forest cover based on these average coefficient estimates. The error bars represent the minimum and maximum coefficient estimates.

**Fig 3:**
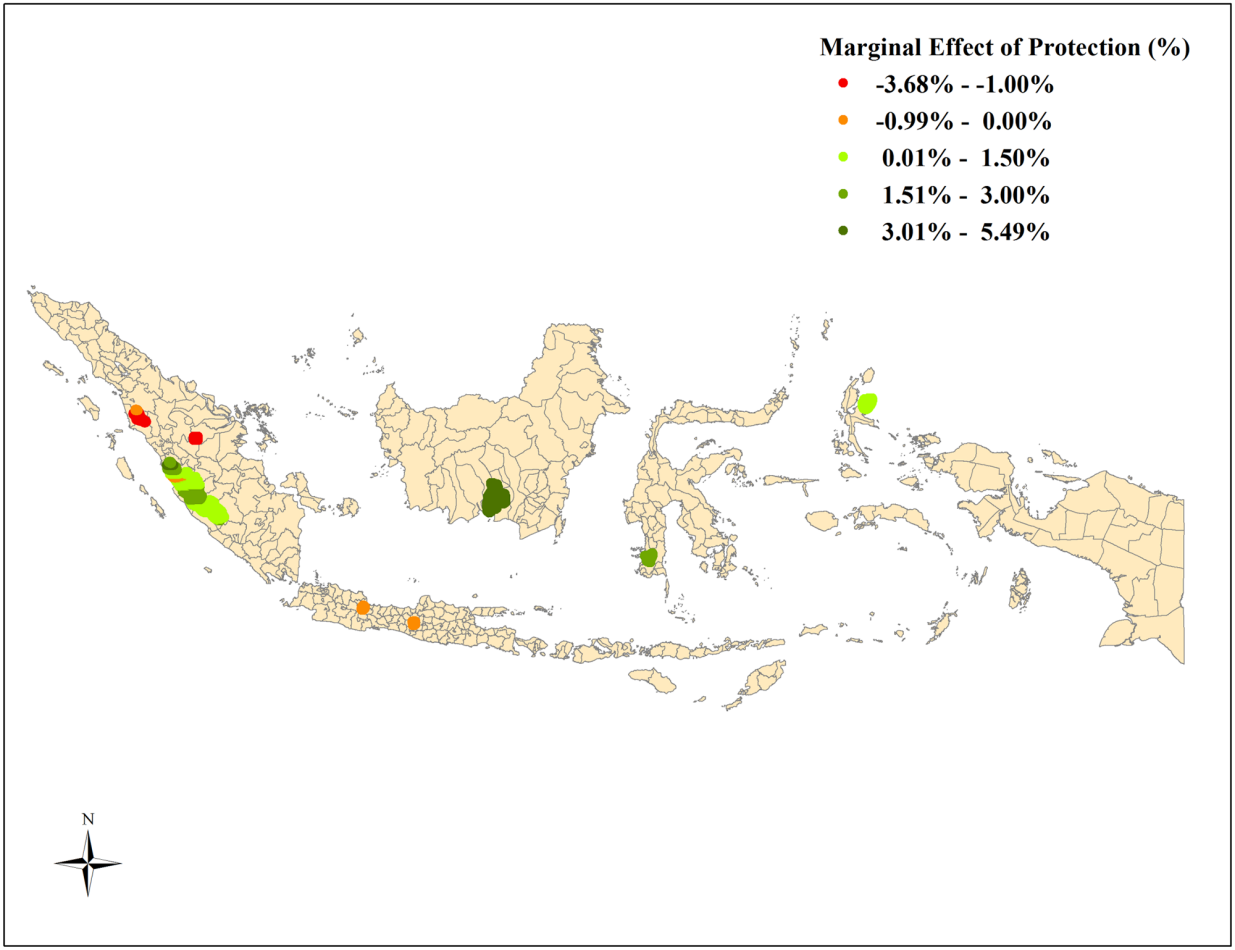
Heterogeneity in Marginal Effect of Protection across the Seven National Parks. Note: This figure maps the coefficient estimates in percentage for the effect of treatment on forest cover change based on the CPARLWR for each parcel with the seven national parks.

To understand the extent of the variation in the effectiveness of protection driven by differences in data generating process, we estimate the median CPARLWR coefficients for the effect of treatment and other covariates that determine forest cover change for the different parks (shown in Table 2). Fig 2 provides a comparison of these coefficient estimates for the seven national parks against the national average for all parks combined. Fig 3 maps the variation in actual coefficient estimates of the effect of protection on change in forest cover across the seven national parks. The median marginal effect of treatment at the national level is a 1.1% increase in forest cover within protected area boundary. The median coefficient estimates for the effect of protection spans a wide range varying from a 5.3% increase in forest cover to a 3.4% decrease in forest cover within protected areas.

The median coefficient estimates for the effect of protection on change in forest cover for Gunung Ciremai, Kerinci Seblat and Aketajawe Lolobata National Parks are similar to the ATT estimates based on the matching analysis. The median marginal effects for Batang Gadis and Tesso Nilo National Park indicate that parcels within protected area boundary witnessed a 1.3% and 3.4% decrease in forest cover. The CPARLWR model is a relatively good fit with a pseudo R^2^ estimate of 26%.

**Fig 4:**
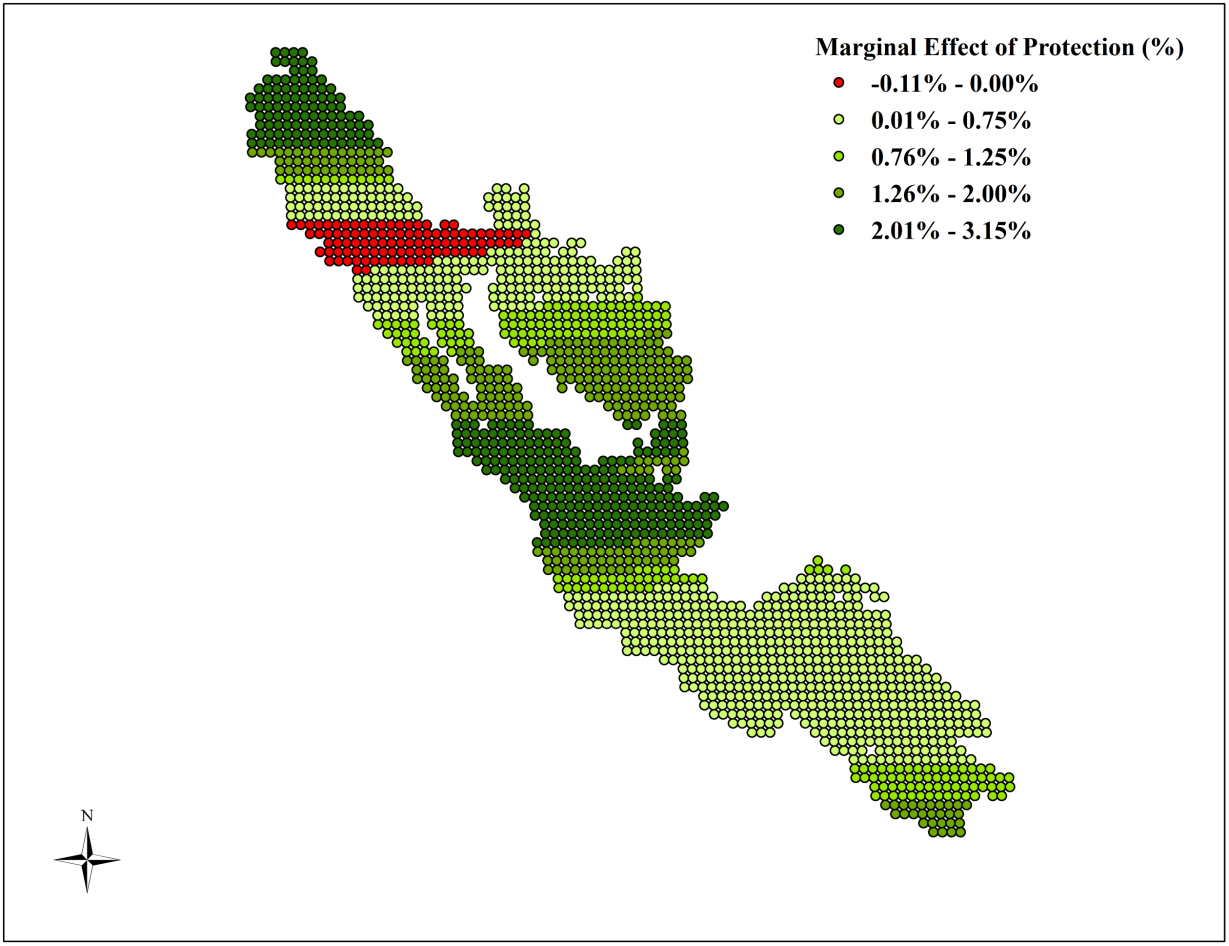
Heterogeneity in Marginal Effect of Protection within Kerinci Seblat National Park. Note: This figures maps the coefficient estimates for the marginal effect of protection on the percentage change in forest cover across Kerinci Seblat National Park based on CPARLWR.

We observe substantial variation in the marginal effects within large parks such as Kerinci Seblat (as shown in Fig 4). While the median marginal coefficient estimate of protection for Kerinci Seblat National Park indicates a small increase in forest cover of 0.73%, certain parts of the park are found to be more effective in increasing forest cover by as much as 3.15%. Fig 4 illustrates the variation in the marginal effects of protection for each parcel for Kerinci Seblat National Park.

**Fig 5:**
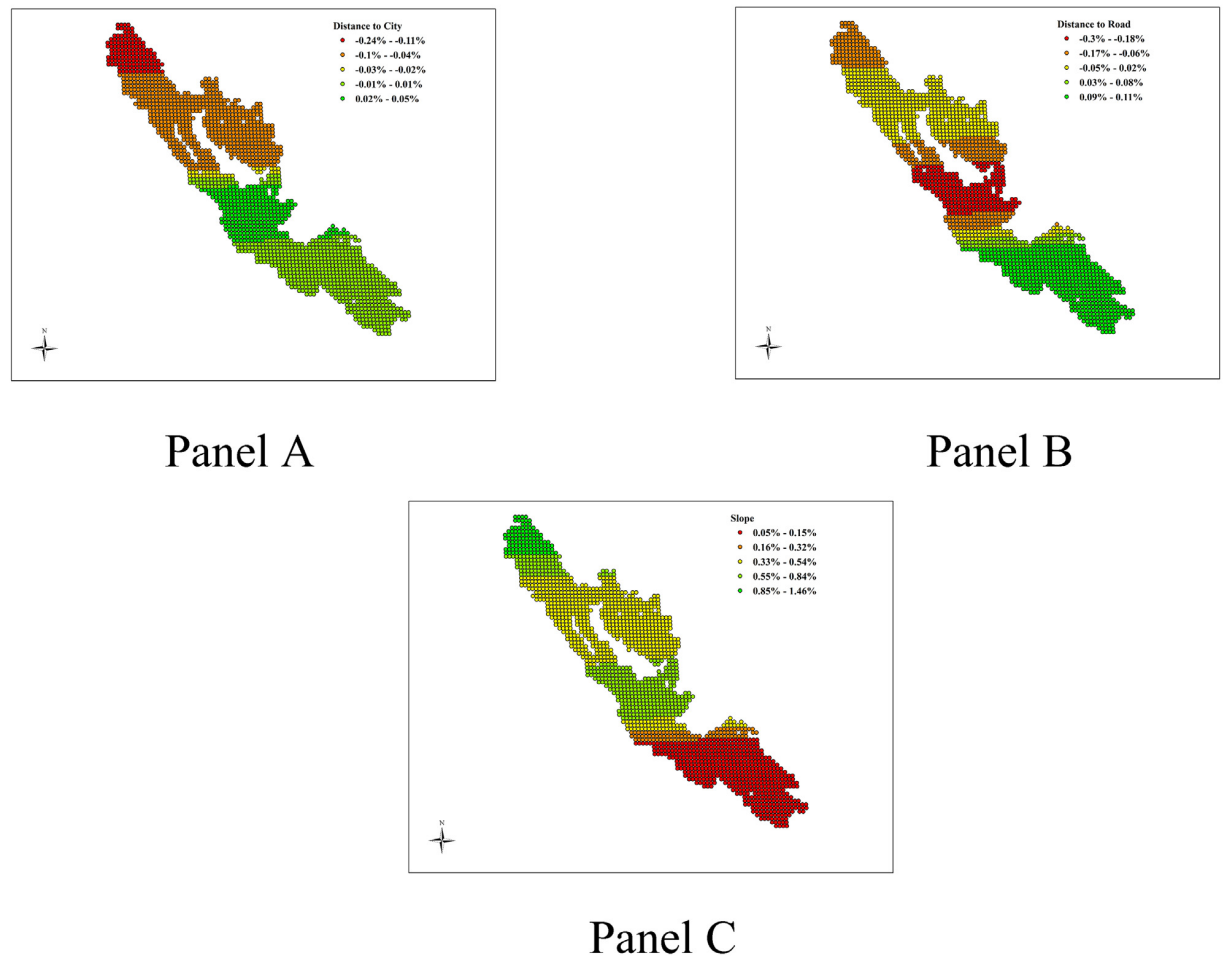
Heterogeneous Impacts of Covariates on Forest Cover Change for Kerinci Seblat National Park. Note: This figure maps the coefficient estimates for the marginal effect of distance to city (Panel A), distance to road (Panel B) and slope (Panel C) for Kerinci Seblat National Park based on CPARLWR.

The CPARLWR model includes all the covariates that we use in the matching process. In Fig 5, we illustrate the heterogeneity in the marginal effects of three covariates, distance to city, distance to road and slope on forest cover change across Kerinci Seblat National Park. In the southern part of the park, we find that the marginal effect of distance from city is positive (as shown in Fig 5 Panel A). Thus protected areas at larger distance from city are shown to exhibit less forest cover loss. The reverse is found in the northern part of the park where we find negative marginal effects of distance from city on forest cover change. Some of these areas also overlap with the parts of the park where we find negative treatment effect. These areas may be prone to increased illegal activities and the distance from city reduces the extent of monitoring and enforcement on these areas and in turn enhances the illegal deforestation activities. The marginal effect of distance to road leads to more forest cover in the southern most parts of the park but reduces forest cover in the central and northern most parts (as shown in Fig 5, Panel B). The marginal effect of slope is always positive, though the effect is substantially larger in the northern most parts of the park and substantially lower in the southern most parts (as shown in Fig 5, Panel C).

## Discussion and Conclusion

In this paper we estimate the effect of protection in seven national parks established between 1999 and 2012, and the variation in these effects across Indonesia. Using a range of methods, we estimate that on average, national parks preserved between 0.01% and 1.12% of forest cover. Regardless of the estimation strategy, however, we find substantial variation in the effect from park to park.

Results from the nearest neighbor matching estimation using controls selected by spatial matching indicate that protected areas have been moderately successful in conserving forests in Indonesia. The ATT estimate for Indonesia of 0.7% is robust to several specification biases such as elimination of parcels adjacent to protected areas as controls and caliper matching. Given the total loss of primary forest cover of 6.2% over this time, preserving 0.7% of forest cover decreases the loss by more than one-tenth at least within the seven new protected areas. We find that an estimated 20,000 hectares of forests across Indonesia would have been lost in the absence of the seven new protected areas.

When we estimate the impact of protection in each of the seven new national parks, we find that the effectiveness of individual parks is significantly different than the national ATT estimates. The estimated levels of effectiveness for four parks, Batang Gadis, Kerinci Seblat, Bantimurung Bulusaraung and Aketajawe Lolobata are less sensitive to the possibility of spurious variation in ATT estimates driven by the effect of unobservables. We find Sebangau National Park is relatively highly effective in protecting against local demands for land conversion but its effect is smaller when compared to losses in unprotected areas that restrict outright conversion but allow for commercial forestry activities.

Our results based on the CPARLWR approach that removes possible spatially correlated hidden bias indicate that Sebangau and Bantimurung Bulusaraung National Park are more effective than the national average estimate in reducing deforestation. Sebangau National Park’s average marginal effect of protection is 4.1% higher than the national estimate based on CPARLWR. The results for Sebangau National Park are more optimistic about the effect of protected areas in Kalimantan given that previous studies [22] had raised substantial concerns over the increase in illegal logging activities in some of the previously established parks in the region such as Tanjung Putting National Park. We find that two Sumatran national parks, Tesso Nilo and Batang Gadis National Park are ineffective in reducing deforestation pressures. These results are similar to findings by [19] which also indicate that protected areas in Sumatra are not very effective, especially when compared with unprotected parcels that allow for sustainable forest use but where outright conversion is limited.

In the case of large parks such as Kerinci Seblat, this method enables us to identify substantial variation in effectiveness even within the park. Our results illustrate that some parts of Kerinci Seblat National Park are indeed effective in reducing deforestation whereas other parts of the park experience a small increase in deforestation. Thus, evaluating the impact of protection at a wider scale is not adequate to identify more local effects. The heterogeneity in forest cover change patterns across protected areas in Indonesia also suggest that rather than a cookie-cutter approach to protection, future protection policy in Indonesia can benefit from more area specific approaches. For example, the decrease in forest cover within certain parts of Kerinci Seblat National Park may signal the possible need for greater resources for monitoring and enforcement of protected areas in this region. More detailed research that focuses on different parts of Kerinci Seblat National Park can help identify why protection is moderately effective in some areas but ineffective in others.

Across all specifications and methods, we find that Sebangau appears to be more effective than the national average while Teso Nilo and Gunung Cirimai appear to be less effective. With other parks, it is less clear whether the estimated differences in effectiveness are driven by differing degrees of bias in the estimates. These results may point to the need for future research that explores the cause of these regional differences and can lead to policy changes that can expand the success or explicitly address the cause of the failure in protection.

One limitation of this study is we only consider areas designated as protected after 2000. Setting aside land for protection in Indonesia is not a recent phenomenon. Indonesia has a long history of protected area management with the biggest thrust occurring in the 1980s. Approximately 10% of the current terrestrial area under protection in Indonesia was brought under protection after 1980. A better estimate of the impact of protection should include these protected areas in the impact evaluation measure. However, this would require data on forest cover prior to 1980. If such data were available, one could calculate more robust ATT estimates using a panel data set and use the larger sample of parks to better explore why some succeeded and others failed. The availability of forest cover data from such an early period would also help control for any underlying trends in deforestation.

Our results for individual parks are unbiased to the extent that the covariates included in the matching process are relevant and any other unobserved variables that significantly affect selection of protected areas and deforestation decisions are spatially correlated. However, if unobservable variables signify distinct spatial discontinuities in selection of protected areas and/or the decision to deforest, the marginal effect of protection based on our CPARLWR estimates will be biased. Note that effectively the CPARLWR uses nearby matched plots as controls, assuming that the unobservables and their effect will be similar across space. Because of this assumption, one might also worry that substantial nearby forest leakage might artificially inflate our CPARLWR treatment estimates.

In this article we present a systematic method for evaluating estimates of program heterogeneity across space. Given that average effects of protection may be misleading, we believe that the move to estimating heterogeneous effects should be encouraged, while we also demonstrate that it should be approached with some caution. Future work that looks at the key drivers of this heterogeneity in impacts of protection in parks across Indonesia can help policy makers identify those characteristics that are associated with greater and less success, and use this information to both target protected areas and enforcement accordingly.

## Supporting Information

S1 Table Summary Statistic for Parcel Level Data Before Matching. Note: ^**1**^This includes seven national parks established in Indonesia in 2004 (Gunung Ciremai, Batang Gadis, Tesso Nilo, Sebangau, Bantimurung Bulusaraung and Aketajawe Lolobata) and one national park established in 1999 (Kerinci Seblat). Two additional national parks (Gunung Merbabu and Gunung Merapi) were established in Java between 2000 and 2012. However, we do not include data associated with these two national parks because of their small size and imprecise forest cover data.
(DOCX)

S2 Table Estimated Change in Forest Cover: Non-Parametric Results (SE). This result is the bias adjusted ATT estimate based on a nearest neighbor covariate matching in which we include parcel level own covariates only.This result is a bias adjusted ATT estimate based on a matching analysis in which we include parcel level own covariates as well as spatially lagged values of neighboring parcels’ covariates.This result is a bias adjusted ATT estimate based on a matching analysis in which we include parcel level own covariates as well as spatially lagged values of neighboring parcels’ covariates but where we drop all parcels that are immediately adjacent to the protected area boundary.This result is a bias adjusted ATT estimate based on a matching analysis in which we include parcel level own covariates as well as spatially lagged values of neighboring parcels’ covariates but we use calipers to restrict matches to within one standard deviation of each covariate.
(DOCX)

S3 Table Covariate Balance and Γ_2_ for Indonesia. Notes: ^1^ The treated number of observations post matching is different from the pre-matching number of observation of 3057. Fifteen treated parcels from Kerinci Seblat National park were dropped because the matching routine was unable to find relevant counterfactuals that exactly matched the treatment parcels with respect to province and WWF ecoregion category.
^2^ Γ_2_ represents the odds ratio for which which the ATT estimate is not sensitive to possible hidden bias due to influence of unobservable variable(s) on the selection of protected area location. Thus, a higher Γ_2_ implies that the results are still significantly different from zero even at a high possible level of bias.
(DOCX)

S4 Table Covariate Balance and Γ_2_ for Gunung Ciremai National Park. Notes: ^1^ Γ_2_ represents the odds ratio that indicates the extent to which the ATT estimate is not sensitive to possible hidden bias due to influence of unobservable variable(s) on the selection of protected area location.
(DOCX)

S5 Table Covariate Balance and Γ_2_ for Batang Gadis National Park. Notes: ^1^ Γ_2_ represents the odds ratio that indicates the extent to which the ATT estimate is not sensitive to possible hidden bias due to influence of unobservable variable(s) on the selection of protected area location.
(DOCX)

S6 Table Covariate Balance and Γ_2_ for Tesso Nilo National Park. Notes: ^1^ Γ_2_ represents the odds ratio that indicates the extent to which the ATT estimate is not sensitive to possible hidden bias due to influence of unobservable variable(s) on the selection of protected area location.
(DOCX)

S7 Table Covariate Balance and Γ_2_ for Kerinci Seblat National Park. Notes: ^1^ Γ_2_ represents the odds ratio that indicates the extent to which the ATT estimate is not sensitive to possible hidden bias due to influence of unobservable variable(s) on the selection of protected area location.
(DOCX)

S8 Table Covariate Balance and Γ_2_ for Sebangau National Park. Notes: ^1^ Γ_2_ represents the odds ratio that indicates the extent to which the ATT estimate is not sensitive to possible hidden bias due to influence of unobservable variable(s) on the selection of protected area location.
(DOCX)

S9 Table Covariate Balance and Γ_2_ for Bantimurung BuluSaraung National Park. Notes: ^1^ Γ_2_ represents the odds ratio that indicates the extent to which the ATT estimate is not sensitive to possible hidden bias due to influence of unobservable variable(s) on the selection of protected area location.
(DOCX)

S10 Table Covariate Balance and Γ_2_ for Aketajawe Lolobata National Park. Notes: ^1^ Γ_2_ represents the odds ratio that indicates the extent to which the ATT estimate is not sensitive to possible hidden bias due to influence of unobservable variable(s) on the selection of protected area location.
(DOCX)

S1 Text Additional Notes on Methods. (DOCX)

## References

[1] IUCN and UNEP-WCMC. The world database on protected areas (WDPA); 1 2011 Cambridge, UK: UNEP-WCMC Available: www.protectedplanet.net.

[2] Andam KS, Ferraro PJ, Sims KR, Healy A, Holland MB. Protected areas reduced poverty in Costa Rica and Thailand. Proceedings of the National Academy of Sciences, 2010; 107(22), 9996–10001. 10.1073/pnas.0914177107 20498058 PMC2890456

[3] Sims KR. Conservation and development: Evidence from Thai protected areas. Journal of Environmental Economics and Management, 2010; 60(2), 94–114.

[4] Ferraro PJ, Hanauer MM. Protecting ecosystems and alleviating poverty with parks and reserves: ‘win-win’or tradeoffs? Environmental and Resource Economics, 2011; 48(2), 269–286.

[5] Ferraro PJ, Hanauer MM, Sims KR. Conditions associated with protected area success in conservation and poverty reduction. Proceedings of the National Academy of Sciences, 2011; 108(34), 13913–13918. 10.1073/pnas.1011529108 21873177 PMC3161530

[6] Joppa LN, Pfaff A. Global protected area impacts. Proceedings of the Royal Society B: Biological Sciences, 2011; 278(1712), 1633–1638. 10.1098/rspb.2010.1713 21084351 PMC3081759

[7] Nelson A, Chomitz KM. Effectiveness of strict vs. multiple use protected areas in reducing tropical forest fires: a global analysis using matching methods. PLoS One, 2011; 6(8), e22722 10.1371/journal.pone.0022722 21857950 PMC3156699

[8] Honey-Rosés J, Baylis K, Ramirez MI. A spatially explicit estimate of avoided forest loss. Conservation Biology, 2011; 25(5), 1032–1043. 10.1111/j.1523-1739.2011.01729.x 21902720

[9] Ferraro PJ, Pattanayak SK. Money for nothing? A call for empirical evaluation of biodiversity conservation investments. PLoS biology, 2006; 4(4), e105 16602825 10.1371/journal.pbio.0040105 PMC1435411

[10] Linkie M, Smith RJ, Zhu Y, Martyr DJ, Suedmeyer E, Pramono J et al Evaluating biodiversity conservation around a large Sumatran protected area. Conservation Biology, 2008; 22, 683–690. 10.1111/j.1523-1739.2008.00906.x 18336620

[11] Levang P, Sitorus S, Gaveau D, Sunderland T. Landless farmers, sly opportunists, and manipulated voters: the squatters of the Bukit Barisan Selatan National Park (Indonesia). Conservation and Society, 2012; 10(3), 243.

[12] Ferraro PJ. Counterfactual thinking and impact evaluation in environmental policy. New Directions for Evaluation, 2009; 122(2009), 75–84.

[13] Pfaff A, Robalino J, Lima E, Sandoval C Herrera LD. Governance, location and avoided deforestation from protected areas: greater restrictions can have lower impact, due to differences in location. World Development, 2014; 55, 7–20. (10.1016/j.worlddev.2013.01.011)

[14] Haruna A, Pfaff A van den Ende S, Joppa L. Evolving protected-areas impacts in Panama: impact shifts show that plans require anticipation. Environmental Research Letters, 2014; 9(3), 035007 10.1088/1748-6041/9/3/035007 24770899

[15] Pfaff A, Santiago-Avila F, Carnovale M Joppa L. Protected areas' impacts upon land cover within Mexico: the need to add politics and dynamics to static land-use economics Agricultural and Applied Economics Association Annual Meeting; 2014 7 27–29; Minneapolis, Minnesota. No. 177195.

[16] Pfaff A, Robalino J. Protecting forests, biodiversity and the climate: predicting policy impact to improve policy choice. Oxford Review of Economic Policy, 2012; 28(1):164–179.

[17] Pfaff A, Amacher GS, Sills EO. Realistic REDD: improving the forest impacts of domestic policies in different settings. Review of Environmental Economics and Policy, 2013; 7(1):114–135 (10.1093/reep/res023).

[18] Andam KS, Ferraro PJ, Pfaff A, Sanchez-Azofeifa GA, Robalino JA. Measuring the effectiveness of protected area networks in reducing deforestation. Proceedings of the National Academy of Sciences, 2008; 105(42), 16089–16094. 10.1073/pnas.0800437105 18854414 PMC2567237

[19] Gaveau DLA, Curran LM, Paoli GD, Carlson KM, Wells P, Besse-Rimba A, et al Examining protected area effectiveness in Sumatra: importance of regulations governing unprotected lands. Conservation Letters, 2012; 5(2), 142–148.

[20] Gaveau DL, Epting J, Lyne O, Linkie M, Kumara I, Kanninen M, et al Evaluating whether protected areas reduce tropical deforestation in Sumatra. Journal of Biogeography, 2009; 36(11), 2165–2175.

[21] Sunderlin WD, Resosudarmo IAP. Rates and causes of deforestation in Indonesia: towards a resolution of the ambiguities. CIFOR, 1996; CIFOR Occasional Paper no. 9(E) (10.17528/cifor/000056)

[22] Fuller DO, Jessup TC, Salim A. Loss of forest cover in Kalimantan, Indonesia, since the 1997–1998 El Nino. Conservation Biology, 2004; 18(1), 249–254.

[23] Kinnaird MF, Sanderson EW, O'Brien TG, Wibisono HT, Woolmer G. Deforestation trends in a tropical landscape and implications for endangered large mammals. Conservation Biology, 2003; 17(1), 245–257.

[24] Curran LM, Trigg SN, McDonald AK, Astiani D, Hardiono YM, Siregar P et al Lowland forest loss in protected areas of Indonesian Borneo. Science, 2004; 303(5660), 1000–1003. 14963327 10.1126/science.1091714

[25] Rhee S, Kitchener D, Brown T, Merrill R, Dilts R, Tighe S. Report on Biodiversity and tropical forests in Indonesia Prepared for USAID, Indonesia 2004 Available: http://pdf.usaid.gov/pdf_docs/pnada949.pdf

[26] Gaveau DL, Wandono H, Setiabudi F. Three decades of deforestation in southwest Sumatra: Have protected areas halted forest loss and logging, and promoted re-growth? Biological Conservation, 2007; 134(4), 495–504.

[27] Fuller DO, Hardiono M, Meijaard E. Deforestation projections for carbon-rich peat swamp forests of Central Kalimantan, Indonesia. Environmental management, 2011; 48(3), 436–447. 10.1007/s00267-011-9643-2 21359865

[28] Carlson KM, Curran LM, Ratnasari D, Pittman AM, Soares-Filho BS, Asner GP, et al Committed carbon emissions, deforestation, and community land conversion from oil palm plantation expansion in West Kalimantan, Indonesia. Proceedings of the National Academy of Sciences, 2012; 109(19), 7559–7564. 10.1073/pnas.1200452109 22523241 PMC3358834

[29] Cannon CH, Summers M, Harting JR, Kessler PJ. Developing conservation priorities based on forest type, condition, and threats in a poorly known ecoregion: Sulawesi, Indonesia. Biotropica, 2007; 39(6), 747–759.

[30] Wheeler D, Hammer D, Kraft R. FCPR—Forest Conservation Performance Rating for the Pan-Tropics. Center for Global Development Working Paper, 2012; (294).

[31] Jepson P, Jarvie JK, MacKinnon K, Monk KA. The end for Indonesia's lowland forests? Science, 2001; 292(5518), 859–861. 11341279 10.1126/science.1061727

[32] Wardojo W, Masripatin N. Trends in Indonesian forest policy. Policy Trend Report, 2002; 77–87.

[33] Olsen N, Bishop J. The financial costs of REDD: evidence from Brazil and Indonesia, International Union for the Conservation of Nature, Gland, Switzerland 2008.

[34] Atmadja S, Wollenberg E. Indonesia. In: Springate-Baginski O Wollenberg E, editors. REDD, forest governance and rural livelihoods: the emerging agenda. Bogor, Indonesia, Center for International Forestry Research (CIFOR); 2010 p. 73–94.Available: http://www.cifor.org/library/3056/indonesia/?pub=3056.

[35] Burgess R, Hansen M, Olken BA, Potapov P, Sieber S. The political economy of deforestation in the tropics (No. w17417). National Bureau of Economic Research 2011.

[36] Schwarze S, Priess JA, Zeller M. Do National Parks reduce deforestation? The effectiveness of the Lore-Lindu National Park in Indonesia. STORMA Discussion Paper Series, Sub-program A on Social and Economic Dynamics in Rain Forest Margins, Göttingen and Bogor. 2009.

[37] Pfaff A, Robalino J, Sanchez-Azofeifa GA, Andam KS, Ferraro PJ. Park location affects forest protection: Land characteristics cause differences in park impacts across Costa Rica. The BE Journal of Economic Analysis & Policy, 2009; 9(2).

[38] Baylis K, Fullerton D Shah P. What drivers forest leakage? Allied Social Sciences Annual Meeting; 2015 1 3–5; Boston, USA.

[39] Margono BA, Potapov PV, Turubanova S, Stolle F, Hansen MC. Primary forest cover loss in Indonesia over 2000–2012. Nature Climate Change, 2014 25737744

[40] Sanchez-Azofeifa GA, Pfaff A, Robalino JA, Boomhower JP. Costa Rica's payment for environmental services program: intention, implementation, and impact. Conservation Biology, 2007; 21(5), 1165–1173. 17883482 10.1111/j.1523-1739.2007.00751.x

[41] Alix- Garcia JM., Shapiro EN, Sims KR. Forest conservation and slippage: Evidence from Mexico’s national payments for ecosystem services program. Land Economics, 2012; 88(4), 613–638.

[42] Busch J, Grantham HS. Parks versus payments: reconciling divergent policy responses to biodiversity loss and climate change from tropical deforestation. Environmental Research Letters, 2013; 8(3), 034028.

[43] Busch J, Lubowski RN, Godoy F, Steininger M, Yusuf AA, Austin K, et al Structuring economic incentives to reduce emissions from deforestation within Indonesia. Proceedings of the National Academy of Sciences, 2012; 109(4), 1062–1067. 10.1073/pnas.1109034109 22232665 PMC3268282

[44] Gaveau D. Protected areas in Sumatra. ARD Learning Exchange 2012. Forests, Trees and Landscape—Synergy, Tradeoff, Challenges, 2012 Available: http://www.cifor.org/ard/documents/background/Day4.pdf.

[45] Linkie M, Dinata Y, Nugroho A, Haidir IA. Estimating occupancy of a data deficient mammalian species living in tropical rainforests: sun bears in the Kerinci Seblat region, Sumatra. Biological Conservation, 2007; 137(1), 20–27.

[46] Ishii A. Park Buffer Zone Reforestation Initiative: Batang Gadis National Park in Sumatra, Indoneisa [thesis]. Duke University; 2007. Available: http://dukespace.lib.duke.edu/dspace/bitstream/handle/10161/285/MP_ai7_a_052007.pdf?sequence=1.

[47] WWF-Indonesia Palming off a national park. Tracking illegal oil palm fruit in Riau, Sumatra. WWF Report. 2013. Available: http://awsassets.wwf.or.id/downloads/wwf_indonesia_palming_off_a_national_park_final.pdf.

[48] Irawan DE, Puradimaja DJ. The hydrogeology of the volcanic spring belt, east slope of Gunung Ciremai, West Java, Indonesia. Proceeding of IAEG Conference; 2006.

[49] Page SE, Banks CJ, Rieley JO. Tropical peatlands: distribution, extent and carbon storage-uncertainties and knowledge gaps. Peatlands International, 2007; 2(2), 26–27.

[50] Vetter J. Impacts of Deforestation on the Conservation Status of Endemic Birds in the North Maluku Endemic Bird Area from 1990–2003 [dissertation]. Duke University; 2009.

[51] Busch J, Lubowski R, Godoy F, Juhn D, Austin K, Hewson J, et al. Open Source Impacts of REDD+ Incentives Spreadsheet—Indonesia (OSIRIS-Indonesia), 2010; Version 1.5. Available: http://www.conservation.org/osiris.

[52] Ho DE, Imai K, King G, Stuart EA. Matching as nonparametric preprocessing for reducing model dependence in parametric causal inference. Political analysis, 2007; 15(3), 199–236.

[53] Sekhon JS. Multivariate and Propensity Score Matching Software with Automated Balance Optimization: The Matching Package for R. Journal of Statistical Software, 2007; 42 (7). Available: http://sekhon.berkeley.edu/papers/MatchingJSS.pdf. Accessed 2014 Sep 15.

[54] Rosenbaum PR, Rubin DB. The central role of the propensity score in observational studies for causal effects. Biometrika, 1983; 70(1), 41–55.

[55] Imbens GW. Nonparametric estimation of average treatment effects under exogeneity: A review. Review of Economics and statistics, 2004; 86(1), 4–29.

[56] Riswan SW, Jenson J, Refisch J, Nellemann C. The Orangutan and the Economics of Sustainable Forest Management in Sumatra. United Nations Environment Programme, Great Apes Survival Partnership, Pan Eco, Yayasan Ekosistem Lestari, World Agroforestry Centre, GRID-Arendal, 2011. Available: http://www.unep.org/pdf/orangutan_report_scr.pdf.

[57] Angelsen A, Kaimowitz D. Rethinking the causes of deforestation: Lessons from economic models. The World Bank Research Observer, 1999; 14 (1) 73–98. 12322119 10.1093/wbro/14.1.73

[58] Pagiola S. Land use change in Indonesia Background paper prepared for the Environment Department, World Bank, Washington, DC, 2000. Available: file:///C:/Users/psshah81/Downloads/0405007.pdf.

[59] Alix-Garcia J. A spatial analysis of common property deforestation.*Journal of* Environmental Economics and Management, 2007; 53(2), 141–157.

[60] Mertens B, Lambin EF. Land-cover-change trajectories in southern Cameroon. Annals of the association of American Geographers, 2000; 90(3), 467–494.

[61] Anselin L. Under the hood issues in the specification and interpretation of spatial regression models. Agricultural economics, 2002; 27(3), 247–267.

[62] Lorena RB, Lambin EF. The spatial dynamic of deforestation and agent use in the Amazon. Applied Geography, 2009; 29:171–181.

[63] Alix-Garcia JM, Shapiro E, Sims KRE. The environmental effectiveness of payments for environmental services in Mexico: Results from a pilot analysis Working paper. University of Wisconsin, Madison, 2010; Available: http://www.aae.wisc.edu/events/papers/devecon/2010/alix-garcia.05.06.pdf

[64] Abadie A, Imbens GW. Large sample properties of matching estimators for average treatment effects. Econometrica, 2006; 74(1), 235–267.

[65] Imbens G, Wooldridge JM. New developments in econometrics. Lecture Notes, CEMMAP, UCL2009b, 2009.

[66] Ho DE, Imai K, King G, Stuart EA. MatchIt: Nonparametric preprocessing for parametric causal inference. Journal of Statistical Software, 2009; 42(8), 1–28.

[67] Rosenbaum PR. Sensitivity to hidden bias Observational studies. Springer, New York; 2002.

[68] Jacoby WG. Loess: a nonparametric, graphical tool for depicting relationships between variables. Electoral Studies, 2000; 19(4), 577–613.

[69] McMillen D Soppelsa ME. A conditionally parametric probit model of micro-data land use in Chicago. Journal of Regional Science, 2014. (10.1111/jors.12174).

