# Supplementary material for: Evaluating Heterogeneous Conservation Effects of Forest Protection in Indonesia
Source: PLoS One. 2015 Jun 3;10(6):e0124872. doi: 10.1371/journal.pone.0124872 (PMC4454437; doi:10.1371/journal.pone.0124872)
Supplement: S1 Table — Note: 1This includes seven national parks established in Indonesia in 2004 (Gunung Ciremai, Batang Gadis, Tesso Nilo, Sebangau, Bantimurung Bulusaraung and Aketajawe Lolobata) and one national park established in 1999 (Kerinci Seblat). Two additional national parks (Gunung Merbabu and Gunung Merapi) were established in Java between 2000 and 2012. However, we do not include data associated with these two national parks because of their small size and imprecise forest cover data. (DOCX) [file pone.0124872.s001.docx]

**S1 Table: Summary Statistic for Parcel Level Data Before Matching**

| Variable | Mean Treated Parcels | Mean Possible Control Parcels |  | Normalized Difference | Mean Raw eQQ Difference |
| --- | --- | --- | --- | --- | --- |
| Forest Cover Change (ha) | -32.94 | -31.40 |  | 0.01 | 2.74 |
| Forest Cover in 2000 (ha) | 750.58 | 441.88 |  | 0.64 | 308.7 |
| Peatland (tC/ha) | 896.72 | 174.16 |  | 0.32 | 721.36 |
| Distance City (km) | 74.68 | 126.12 |  | -0.41 | 54.26 |
| Distance River (m) | 3651.66 | 3244.35 |  | 0.09 | 530.68 |
| Distance Road (km) | 31.23 | 59.13 |  | -0.31 | 30.78 |
| Elevation (m) | 712.32 | 304.23 |  | 0.54 | 426.39 |
| Slope (degree) | 11.94 | 6.84 |  | 0.50 | 5.20 |
| W Forest Cover in 2000 (ha) | 736.3 | 435.9 |  | 0.69 | 300.4 |
| W Peatland (tC/ha) | 892.12 | 170.52 |  | 0.33 | 720.47 |
| W Distance City (km) | 74.40 | 123.26 |  | -0.39 | 51.92 |
| W Distance River (m) | 3617.98 | 3128.29 |  | 0.12 | 568.52 |
| W Distance Road (km) | 31.10 | 57.38 |  | -0.30 | 29.27 |
| W Elevation (m) | 702.15 | 301.90 |  | 0.57 | 414.44 |
| W Slope (deg.) | 11.75 | 6.75 |  | 0.53 | 5.22 |
| N Treated | 3,057^1^ | | | | |
| N Available Controls | 170,878 | | | | |
