# Supplementary material for: Evaluating Heterogeneous Conservation Effects of Forest Protection in Indonesia
Source: PLoS One. 2015 Jun 3;10(6):e0124872. doi: 10.1371/journal.pone.0124872 (PMC4454437; doi:10.1371/journal.pone.0124872)
Supplement: S2 Table — This result is the bias adjusted ATT estimate based on a nearest neighbor covariate matching in which we include parcel level own covariates only.This result is a bias adjusted ATT estimate based on a matching analysis in which we include parcel level own covariates as well as spatially lagged values of neighboring parcels’ covariates.This result is a bias adjusted ATT estimate based on a matching analysis in which we include parcel level own covariates as well as spatially lagged values of neighboring parcels’ covariates but where we drop all parcels that are immediately adjacent to the protected area boundary.This result is a bias adjusted ATT estimate based on a matching analysis in which we include parcel level own covariates as well as spatially lagged values of neighboring parcels’ covariates but we use calipers to restrict matches to within one standard deviation of each covariate. (DOCX) [file pone.0124872.s002.docx]

**S2 Table: Estimated Change in Forest Cover: Non-Parametric Results (robust SE)**

| **ATT Estimate in % for Different Matching Routines** | |
| --- | --- |
|  |  |
| **Matching^a^** | 2.58% |
|  | (0.53%) |
| **Spatial Matching I^b^** | 0.71% |
|  | (0.43%) |
| **Spatial Matching II (without 6km boundary)^c^** | 0.18% |
|  | (0.54%) |
| **Spatial Matching I with Calipers^d^** | 0.50% %) |
|  | (0.29 |
