# Supplementary material for: Evaluating Heterogeneous Conservation Effects of Forest Protection in Indonesia
Source: PLoS One. 2015 Jun 3;10(6):e0124872. doi: 10.1371/journal.pone.0124872 (PMC4454437; doi:10.1371/journal.pone.0124872)
Supplement: S3 Table — Notes: 1 The treated number of observations post matching is different from the pre-matching number of observation of 3057. Fifteen treated parcels from Kerinci Seblat National park were dropped because the matching routine was unable to find relevant counterfactuals that exactly matched the treatment parcels with respect to province and WWF ecoregion category. 2 Γ2 represents the odds ratio for which which the ATT estimate is not sensitive to possible hidden bias due to influence of unobservable variable(s) on the selection of protected area location. Thus, a higher Γ2 implies that the results are still significantly different from zero even at a high possible level of bias. (DOCX) [file pone.0124872.s003.docx]

**S3 Table: Covariate Balance and Γ_2_** **for Indonesia**

| Variable | Mean Treated Parcels | Mean Control Parcels |  | Normalized Difference | Mean Raw eQQ Difference |
| --- | --- | --- | --- | --- | --- |
| Forest Cover in 2000 (ha) | 749.84 | 669.40 |  | 0.19 | 80.45 |
| Peatland (tC/ha) | 901.14 | 733.94 |  | 0.06 | 167.24 |
| Distance City (km) | 74.93 | 77.03 |  | 0.02 | 4.31 |
| Distance River (m) | 3659.08 | 3137.31 |  | 0.12 | 523.48 |
| Distance Road (km) | 31.34 | 27.69 |  | 0.09 | 4.88 |
| Elevation (m) | 710.83 | 659.95 |  | 0.06 | 53.33 |
| Slope (degree) | 11.91 | 11.02 |  | 0.08 | 0.93 |
| W Forest Cover in 2000 (ha) | 735.51 | 647.24 |  | 0.25 | 88.27 |
| W Peatland (tC/ha) | 896.52 | 728.36 |  | 0.06 | 168.17 |
| W Distance City (km) | 74.65 | 76.78 |  | 0.02 | 4.30 |
| W Distance River (m) | 3624.97 | 3160.89 |  | 0.13 | 466.11 |
| W Distance Road (km) | 31.21 | 27.58 |  | 0.09 | 4.86 |
| W Elevation (m) | 700.63 | 650.84 |  | 0.07 | 52.31 |
| W Slope (deg.) | 11.72 | 10.86 |  | 0.09 | 0.90 |
| N Treated | 3042^1^ | | | | |
| N Available Controls | 15210 | | | | |
| Γ_2_^2^ | 1.7 | | | | |
