# Supplementary material for: Evaluating Heterogeneous Conservation Effects of Forest Protection in Indonesia
Source: PLoS One. 2015 Jun 3;10(6):e0124872. doi: 10.1371/journal.pone.0124872 (PMC4454437; doi:10.1371/journal.pone.0124872)
Supplement: S4 Table — Notes: 1 Γ2 represents the odds ratio that indicates the extent to which the ATT estimate is not sensitive to possible hidden bias due to influence of unobservable variable(s) on the selection of protected area location. (DOCX) [file pone.0124872.s004.docx]

**S4 Table: Covariate Balance and Γ_2_ for Gunung Ciremai National Park**

| Variable | Mean Treated Parcels | Mean Control Parcels |  | Normalized Difference | Mean Raw eQQ Difference |
| --- | --- | --- | --- | --- | --- |
| Forest Cover in 2000 (ha) | 210.87 | 159.74 |  | 0.13 | 51.13 |
| Peatland (tC/ha) | 0.00 | 0.00 |  | 0.00 | 0 |
| Distance City (km) | 16.71 | 12.66 |  | 0.49 | 5.18 |
| Distance River (m) | 2733.86 | 2509.18 |  | 0.09 | 280.34 |
| Distance Road (km) | 5.71 | 4.68 |  | 0.21 | 1.86 |
| Elevation (m) | 1090.34 | 950.35 |  | 0.21 | 141.80 |
| Slope (degree) | 13.37 | 11.56 |  | 0.26 | 1.81 |
| W Forest Cover in 2000 (ha) | 191.94 | 146.88 |  | 0.20 | 45.07 |
| W Peatland (tC/ha) | 0.00 | 0.00 |  | 0.00 | 0 |
| W Distance City (km) | 16.54 | 12.57 |  | 0.49 | 5.03 |
| W Distance River (m) | 2865.89 | 2682.74 |  | 0.10 | 308.70 |
| W Distance Road (km) | 5.27 | 4.67 |  | 0.13 | 1.73 |
| W Elevation (m) | 1022.19 | 904.61 |  | 0.24 | 119.43 |
| W Slope (deg.) | 12.35 | 10.75 |  | 0.34 | 1.60 |
| N Treated | 33 | | | | |
| N Available Controls | 165 | | | | |
| Γ_2_^1^ | 1 | | | | |
