# Supplementary material for: Evaluating Heterogeneous Conservation Effects of Forest Protection in Indonesia
Source: PLoS One. 2015 Jun 3;10(6):e0124872. doi: 10.1371/journal.pone.0124872 (PMC4454437; doi:10.1371/journal.pone.0124872)
Supplement: S1 Text — (DOCX) [file pone.0124872.s011.docx]

**S1 Text: Additional Notes on Methods**

We explore the degree of spatial autocorrelation in our dependent variable using a Moran’s I index. The Moran’s I index provides a measure of local homogeneity in the outcome of interest. Its value ranges between -1 to +1 where -1 indicates strong negative spatial autocorrelation and +1 indicates strong positive spatial autocorrelation. To assess how the measure of spatial autocorrelation changes with alternative weights matrices, we also calculate the Moran’s I for second-order and third-order queen’s contiguity weights matrices. Because our data are a grid, contiguity-based definition of neighbors is the same as using a distance based definition or a k nearest neighbor definition -the three most frequently used weights matrices. Thus, to test alternative definitions, we use higher order contiguity matrices, which include more rings of neighboring grids as neighbors. This approach effectively tests how quickly the spatial correlation degrades.

One way to control for spatial autocorrelation is to use a locally-weighted regression. The CPARLWR procedure uses local parametric regressions to allow the data-generating processes to vary smoothly over space without forcing a specific spatial weights structure that assumes a global trend [69]. [69] illustrates the use of a conditionally parametric spatial probit model for a large data set to model residential land use for Chicago. Their results illustrate the benefits of using this flexible functional specification for scenarios where the underlying functional form is complex and/or other forms of misspecification are assumed to vary smoothly over space.

We could alternatively use a spatial lag model that accounts for the spatial effects using a global trend under the assumption that spatial variation around this global trend are relatively small. However, for the spatial lag model, we need to use a deterministic weights matrix that imposes a specific form of spatial relationship between each pair of observations that is assumed to be globally applicable across the whole dataset. The CPARLWR approach includes the spatial information without requiring us to know the true form of the spatial relationship *a priori*.
